# The burden and characteristics of tuberculosis/human immunodeficiency virus (TB/HIV) in South Korea: a study from a population database and a survey

**DOI:** 10.1186/1471-2334-10-66

**Published:** 2010-03-12

**Authors:** Chang-Hoon Lee, Ji-young Hwang, Dae-Kyu Oh, Mee-Kyung Kee, Eunjung Oh, Jung-wook An, Jinhyun Kim, Heonsook Do, Hee-Jin Kim, Sung Soon Kim, Hwahyun Kim, Jeong-Gu Nam

**Affiliations:** 1 Division of HIV and TB Control, Korea Centers for Disease Control and Prevention, 194 Tongillo, Eunpyung-gu, Seoul, 122-701, Republic of Korea; 2 Division of pulmonary and critical care medicine, Department of Internal Medicine, Seoul Metropolitan Government Seoul National University Boramae Medical Center, 39 Boramae Road, Dongjak-gu, Seoul, 156-707, Republic of Korea; 3 Preventive Medicine, Gachon University of Medicine and Science, 534-2 Yeonsu3-dong, Yeonsu-gu, Incheon, 406-799, Republic of Korea; 4 Division of AIDS, Korea Centers for Disease Control and Prevention, 194 Tongillo, Eunpyung-gu, Seoul, 122-701, Republic of Korea; 5 Division of Rheumatology, Department of Internal Medicine, Seoul National University College of Medicine, 28 Yongon-dong, Jongno-gu, Seoul, 110-744, Republic of Korea; 6 Korean Institute of Tuberculosis, Republic of Korea, 14 Woomyeon-dong, Seocho-gu, Seoul, 137-900, Republic of Korea

## Abstract

**Background:**

Although, in South Korea, human immunodeficiency virus/acquired immunodeficiency syndrome(HIV/AIDS) keeps increasing and tuberculosis(TB) burden is still significant, there have been few reports on TB/HIV cases. In this study, we investigated the burden and characteristics of TB/HIV patients in South Korea, an area with intermediate burden of TB and a low prevalent area with HIV/AIDS.

**Methods:**

We identified patients with TB and cases with HIV between January 1 2001 and December 31 2005, from nationwide reporting system (TBnet and HIV/AIDS registry) through an electronic record linkage method. A questionnaire survey was also conducted and determined the rate of diagnosis of HIV among TB cases in public health units in 2005.

**Results:**

The number of cases with both HIV and TB was 137 (0.07% among 197,562 TB cases) and the newly detected TB/HIV cases per 100,000 population was increasing annually: 2001, 0.025; 2002, 0.031; 2003, 0.025; 2004, 0.071; 2005, 0.095. Males between 20 and 59 years of age accounted for 87.6% of TB/HIV patients. Compared with patients with TB alone, those with TB/HIV had a higher percentage of extrapulmonary TB (8.0% vs 19.0%; p < 0.0001). The standardized prevalence ratio (SPR) of HIV among patients with TB was 18.46 (95% CI, 15.50-21.83). SPR of HIV among male TB patients aged 20-59 and extrapulmonary TB cases was 39.64 (95% CI, 32.87-47.40) and 43.21 (95% CI, 28.22-63.31) respectively. Through a questionnaire survey of public health units, six patients (0.08%) were confirmed as having HIV among 7,871 TB patients in public health centers in 2005, which is similar to the result from the study through nationwide reporting systems.

**Conclusions:**

The prevalence rate of TB/HIV patients is still low but increasing in South Korea. Physicians should consider performing HIV tests among TB patients, especially in higher-risk groups, such as young males with extrapulmonary TB in South Korea.

## Background

Tuberculosis (TB) and human immunodeficiency virus/acquired immunodeficiency syndrome (HIV/AIDS) are mankind's major infectious diseases [1,2]. Comorbidity with HIV and TB has been highlighted recently. It is well known that TB and HIV are closely associated. HIV infection contributes to the progression from a recently acquired [3] or latent [4] TB infection to the active form of the disease. HIV infection also increases the risk of recurrence of TB [5]. In addition, the relationship between HIV and drug-resistant TB, including extensively drug resistant (XDR) TB, is cause for concern [6-8]. Conversely, it has been reported that TB may also promote infection [9] with HIV and progression to AIDS [10,11].

According to the World Health Organization, the number of TB cases among HIV-infected people was 0.7 million in 2006, [1] which is significant because HIV is thought to contribute to TB epidemics, especially in sub-Saharan Africa [12]. It was also reported that treatment of latent TB infection could also prevent TB development in TB/HIV cases [13]. In addition, early detection and intervention for HIV among TB cases are also emphasized [14,15]. The World Health Organization stressed that HIV prevention should be a priority for strategies aimed at controlling TB [16] and that all TB patients should be offered HIV counseling and testing [1,17]. However, there could be controversies, because screening tests for HIV have a high rate of false-positive results in countries with low prevalence rate with HIV/AIDS [18]. In addition, how many TB patients underwent HIV testing and how many HIV/AIDS cases were detected among them in individual countries including South Korea has not been fully investigated [19].

It was reported that HIV/AIDS cases have increased in South Korea since the first case was identified in 1985, in spite of the low prevalence rate under 0.01% [20,21]. Although results from seven nationwide prevalence surveys at five-yearly intervals from 1965 to 1995 revealed a significant decrease in the prevalence of TB [22], South Korea is still regarded as a country with intermediate TB burden; however, studies on the burden and characteristics of TB/HIV cases in South Korea has been rarely reported [1]. The purposes of this study were to document the burden and characteristics of TB/HIV patients in Korea, which is an area with low HIV/AIDS prevalence [2] and a country with an intermediate TB burden [1], and to evaluate the contribution of HIV testing among TB patients, by using linkage of national registries, and a survey of public health centers (PHC).

## Methods

### Data sources

The population database for this study consists of three components: Korea TB Surveillance System (KTBS) data, the national registry data of patients with HIV/AIDS (the HIV/AIDS national registry), and data from Korea National Statistical Office (KNSO). In South Korea, the TB prevention law requires doctors to notify regional public health centers of all cases of TB (In the study, TB refers to 'active TB disease'). Additionally, patients suspicious for having HIV infection (for example, cases with positive anti-HIV antibody testing) undergo confirmatory diagnostic testing using the Western blot method at government institutes and all people diagnosed with HIV/AIDS are registered for monitoring. We used the KTBS and the HIV/AIDS national registry to identify people who were diagnosed with HIV/AIDS and TB between 1 January 2001 and 31 December 2005. Comorbidity with HIV/AIDS and TB was verified by identifying patients who had been registered both in KTBS and HIV/AIDS national registry using an electronic record linkage method with Standard Query Language (SQL) procedure with SAS version 9.1 (SAS Institute, NC, USA). The year of comorbidity was determined to be the year when the later infection between TB and HIV/AIDS was diagnosed. Demographic data for 2003 were obtained from the KNSO. [23] and were used to characterize the general population at the midpoint between 2001 and 2005. A questionnaire survey was conducted among managers of PHC, which are infrastructures of Korean public health care system, to determine the number of HIV screening tests conducted among TB patients and the rate of diagnosis of HIV in 2005. This study was not submit to an ethics institutional review board as the data we used for this study is existing data including a questionnaire survey that participants cannot be identified, and the dataset is already reviewed by some authorities of Korea Center for Disease Control and Prevention (KCDC) and Korean Institute of Tuberculosis (KIT).

### Statistical analyses

We characterized demographic data according to age/age group (0-9, 10-19, 20-29, 30-39, 40-49, 50-59, ≥ 60 years of age), sex, date of HIV diagnosis, date of initial anti-TB treatment, CD4+ lymphocyte cell count (data was expressed as median with interquartile range (IQL) upon diagnosis of HIV, and type of TB from the data of population database.

To compare the prevalence rates of HIV infection among TB patients and HIV/AIDS infection among the general Korean population, standardized prevalence ratios (SPRs) [24,25] that were adjusted by sex and age were calculated as:

The expected number of people with HIV was extrapolated from the prevalence rate of HIV among the survivors of the general population at 30^th ^June, 2003 (the midpoint of the study), which was based on the register of HIV/AIDS and the KNSO. For example, 'SPR of HIV is 5.0 at male TB group' means that the number of observed HIV cases is 5.0 times more than the expected number of HIV cases calculated from the prevalence rate in the general population of men.

The statistical analyses were carried out using the SQL procedures in SAS version 9.1, STATA version 10.1 (StataCorp, Tex, USA) and Microsoft Excel and Powerpoint program (Microsoft, WA, USA)

## Results

**Figure 1:**
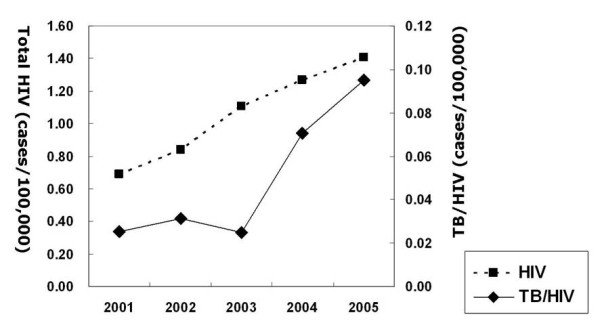
**The trends of newly detected HIV infection cases and TB/HIV cases in South Korea (2001-2005)**.

The total number of patients with HIV/AIDS between 1st January, 2001 and 31th December, 2005 was 2,548. Of these, 92.4% were male. The median age was 37 years (range, 4-80 years). The peak age group was 30-39 years of age (34.3% of all patients). The annual number of newly detected HIV/AIDS cases per 100,000 people increased with time: 2001, 0.69; 2002, 0.84; 2003, 1.11; 2004, 1.27; 2005, 1.41. The average increasing rate of cases with HIV was 19.6% per year (Figure 1). CD4+ lymphocyte cell counts were available for 1,466 patients (57.5% of all confirmed HIV/AIDS patients). The median CD4+ lymphocyte cell count was 315.0 cells/mm^3^(IRQ, 186.0-464.0).

The total number of patients with TB from 1st January, 2001 to 31th December, 2005 was 197,562. Of these, 62.9% were male. The median age was 44 years (range, 0-97 years). Of those less than 60 years of age, the peak age group was 20-29 years old (20.1% of all patients with TB). Most patients had pulmonary TB (92%, including patients with positive sputum smears [36.4%]) and 8% had extrapulmonary TB. The most common type of extrapulmonary TB was TB pleurisy (3.4% of all patients with TB). (Table 1)

**Table 1:** Characteristics of patients with HIV, patients with TB, and patients with coinfection of TB and HIV in Korea (2001-2005).

	HIV (N = 2,548)		TB (N = 197,562)		HIV & TB (N = 137)	
Sex, male : female	2,355(92.4%):193(7.6%)		124,183(62.9%):73,379(37.1%)		132(96.4%):5(3.6%)	

Age, median (range)	37.0 (4-80)		44 (0-97)		39.5 (19.9-79.1)	
0-9	2 (0.1%)		1,000 (0.5%)		0 (0%)	
10-19	60 (2.4%)		12,575 (6.4%)		1 (0.7%)	
20-29	576 (22.6%)		39.638 (20.1%)		19 (13.9%)	
30-39	873 (34.3%)		32,231 (16.3%)		51 (37.2%)	
40-49	583 (22.9%)		30,515 (15.4%)		41 (29.9%)	
50-59	318 (12.5%)		23,206 (11.7%)		14 (10.2%)	
60-	136 (5.3%)		58,397 (29.6%)		11 (8.0%)	

Year	Cases	Incidence (cases/10^5^)	Cases	Incidence (cases/10^5^)	Cases	Incidence (cases/10^5^)

2001	327	0.69	42,737	90.3	17	0.036
2002	398	0.84	38,801	81.4	20	0.042
2003	534	1.11	36,227	75.6	21	0.044
2004	610	1.27	36,041	74.8	40	0.083
2005	680	1.41	43,781	90.7	39	0.081
CD4 count at diagnosis, median(IQR) (/mm^3^)	315.0 (186.0-464.0)				301.0 (171.5-460.0)	

TB type (% among patients with TB)						

Pulmonary			183,640 (92.0%)		111 (81.0%)	
Smear positive			72,599 (36.4%)		42 (30.7%)	
Extrapulmonary			16,021 (8.0%)		26 (19.0%)	
Pleurisy			6,852 (3.4%)		3 (2.2%)	
Lymphnode			3,675 (1.8%)		10 (7.3%)	
Gastrointestinal			1,797 (0.9%)		5 (3.6%)	
Nervous			657 (0.3%)		3 (2.2%)	
Skin			272 (0.1%)		1 (0.7%)	
Other organs			2,169 (1.1%)		1 (0.7%)	
Disseminated			599 (0.3%)		3 (2.2%)	

There were 137 TB/HIV patients between 2001 and 2005, and 96.4% of these were male. The median age was 39 years (range, 19-79 years). The peak age group was 30-39 years (37.2% of all patients), and males aged 20-59 accounted for 87.6% of the total TB/HIV cases. Annual newly detected TB/HIV cases per 100,000 population increased with time (p < 0.001): 2001, 0.025; 2002, 0.031; 2003, 0.025; 2004, 0.071; 2005, 0.095. (Figure 1). Among the patients with TB, 0.07% had HIV and 5.4% of the HIV/AIDS patients contracted TB. The number of HIV/AIDS patients who were subsequently diagnosed with TB was greater than the number of patients with TB who subsequently were diagnosed with HIV. The median time interval before HIV patients were registered with TB was 199 days (range, 3-1,455 days); the median time interval before TB patients were diagnosed with HIV was 20 days (range, 1-1,140 days). For patients with comorbidity, the median CD4+ lymphocyte cell count upon diagnosis of HIV was 301.0 cells/mm^3^. There was no significant difference in the CD4+ lymphocyte cell count at diagnosis of HIV between patients with HIV alone and patients coinfected with TB and HIV (p = 0.587 by Mann-Whitney U test). There was no significant difference in the CD4 count at diagnosis of HIV between TB patients who subsequently contracted HIV and HIV patients who subsequently contracted TB (median CD4+ lymphocyte cell count, 316.5 cells/mm^3 ^vs. 268.0 cells/mm^3^; p = 0.706 by Mann-Whitney U test). Compared with patients with TB alone, those with comorbidity had a higher percentage of extrapulmonary TB (OR, 2.69; 95% CI, 1.75-4.12; p < 0.0001) and similar percentage of sputum smear positivity among pulmonary TB (OR, 0.93; 95% CI, 0.63-1.37; p = 0.715). The most common type of extrapulmonary TB in comorbid patients was TB lymphadenitis (7.3% of all coinfection cases) (Table 1).

The SPR of HIV among overall TB patients was 18.46 (95% CI, 15.50-21.83). SPR of HIV among male aged 20-59 was 39.64 (95% CI, 32.87-47.40), while SPR of HIV among male aged ≤ 19 or ≥ 60 and among female was 7.33 (95% CI, 3.78-12.81) and 7.87 (95% CI, 2.54-18.37), respectively. SPR of HIV among cases with extrapulmonary TB was 43.21 (95% CI, 28.22-63.31), while SPR of HIV among cases with pulmonary TB and among semar-positive TB was also 16.09 (95% CI, 13.24-19.38) and 15.40 (95% CI, 11.10-20.82), respectively. (Table 2)

**Table 2:** SPR of HIV among overall cases with TB.

Subgroup	No. of TB cases	No. of HIV cases		PR	95% CI	
				
		Observed*	Expected		Lower	Upper
By sex						
Male	124,183	132	8.21	16.09	13.46	19.08
Female	73,379	5	0.65	7.87	2.54	18.37

By age						
0-9	1,000	0	0.00	0.00	NA	7,800.11
10-19	12,575	1	0.03	34.87	NA	193.99
20-29	39,638	19	1.48	12.84	7.73	20.05
30-39	32,231	51	2.44	20.92	15.57	27.50
40-49	30,515	41	1.98	20.75	14.89	28.16
50-59	23,206	14	1.11	12.58	6.87	21.12
60-	58,397	11	1.02	10.79	5.38	19.30

Male aged 20-59	80,599	120	3.03	39.64	32.87	47.40
Male aged < 19 or ≥ 60	43,584	12	1.64	7.33	3.78	12.81

Pulmonary TB	183,640	111	6.90	16.09	13.24	19.38
Smear-positive TB	72,599	42	2.73	15.40	11.10	20.82
Extrapulmonary TB	16,021	26	0.60	43.21	28.22	63.31

Overall	197,562	85	7.42	18.46	15.50	21.83

In 2005, HIV screening tests were performed on 7,871 (60.4%) of the 13,028 TB patients (27.7% of total notified cases) registered with PHC in South Korea. Of these, six patients (0.08%), five of whom were male, were confirmed as having HIV.

## Discussion

Our study showed that the number of TB/HIV patients was increasing but the percentage of HIV-infected TB patients was low (0.07%) in Korea. The results of questionnaire surveys conducted at PHC were similar (0.08% of TB patients was infected with HIV).

The annual number of HIV patients increased by 15.8% per year between 2001 and 2005. Although the prevalence rate of patients with TB has decreased in Korea during the last four decades, [22] the number of TB patients notified was constant or has even increased during the five years of our study period [26]. As the prevalence rate of HIV/AIDS increases, the number of TB patients may also increase because the peak age for HIV and that of TB overlap considerably. For example, in our study, 51.8% of patients with TB, 79.8% of people living with HIV/AIDS, and 81.0% of TB/HIV patients were between 20 and 49 years of age. In regions with intermediate-to-high burden TB, it is a common finding that the incidence rate of TB is high in young age groups [27,28]. Although there are other key important issues resulting in TB/HIV comorbidity including overlapping risk factors for the transmission of the two diseases such as intravenous drug use [29], which is rarely attributable to transmission of both diseases in South Korea, given that (1) TB is prevalent in young age groups which is probably due to recent infection in regions with intermediate TB burden, (2) HIV/AIDS infection, in which the immune system destruction is well-known risk factor of TB development [3,4], is also concentrated in similar age groups and would be an obstacle in TB control in young age, and (3) if TB in young ages were not controlled properly, the future burden of TB would still be substantial because of reactivation in older ages from the latent TB infection, overlapping age in both diseases might be a potential threat of TB control in South Korea.

The percentage of HIV infection among TB patients (0.07%) is significantly higher, especially among some subgroups, than that among general population in South Korea (0.06%). The SPRs of HIV infection among patients with TB (18.46) were relatively high, which indicated that the risks of having HIV among TB patients were more than 18 times greater than that of the general population. This was comparable to the results of previous studies [27,30,31]. The proportion of HIV infection (0.07%) among TB patients notified in South Korea between 2001-2005, and the proportion of HIV infection (0.08%) diagnosed by screening test among TB patients of PHC in 2005 in our study are also much greater than the prevalence rate of HIV among general Korean population in 2005, which was 6.4 cases per 100,000 population (0.006%). Therefore, HIV screening of TB patients may be worthwhile in settings such as ours. In addition, we wondered if the low prevalence rate of comorbidity with HIV could indicate TB patients with a high risk of HIV infection should be selected for HIV testing. Knowledge of the epidemiological characteristics of patients with comorbidity, which is affected by the prevalence rate of TB and HIV and the route of transmission of HIV infection, would be helpful [27,29,32,33]. In our study, most patients with TB/HIV were young males, who were mainly caused by this group having a higher sexual activity and being at the peak age for HIV. Most patients acquired HIV by sexual contact (more than 98% of all patients [20]), including a relatively high proportion of homosexual contact with HIV-infected persons (reported as 40% [20], but estimated as more than 50%; data not shown) in South Korea. Patients with comorbidity tended to have a greater proportion of extrapulmonary TB than did patients with TB alone. Among patients with pulmonary TB, those infected with HIV had a lower rate of positive sputum smears than did those with TB alone, although statistical significance could not be demonstrated in our cohort. This result agrees with that of a previous report, which showed that, in patients with HIV/AIDS, especially in the late stage of HIV infection, TB is often atypical in presentation, frequently causes extrapulmonary disease, and has low sputum smear positivity in pulmonary TB, which could result in delayed diagnosis of TB [34]. In our study, SPR of HIV among males aged 20-59 was very high as 39.64, while SPR of HIV among male aged ≤ 19 or ≥ 60 and among female was 7.33 and 7.87. SPR of HIV among cases with extrapulmonary TB was 43.21, however, SPR of HIV among cases with pulmonary TB and among smear-positive TB was also 16.09 and 15.40 respectively, although those SPRs were smaller than that among extrapulmonary TB cases. Based on our results, we recommend that physicians should consider performing HIV tests among patients with TB, especially males aged 20-59 and those with extrapulmonary TB in South Korea. However, we could not completely disagree to the recommendation that all TB patients should undergo HIV testing (universal approach) [1,17] because the SPRs of HIV among other groups such as pulmonary TB cases were not low. In our study, if only male TB patients between age 20 and 59 had undergone HIV testing, 17 cases (12.4% of comorbid cases) with TB/HIV could be missed. On the other hand, if HIV screening were restricted to male TB patients aged 20-59 or patients with extrapulmonary TB, 11 cases (8.0% of comorbid cases) could be missed. The guideline of South Korea recommends all TB patients should undergo HIV testing now.

It could also be thought that the high SPR of HIV among patients with TB suggests that TB increases the risk of contracting HIV, which was, to our knowledge, the first clinical suggestion. Some researchers reported that patients with TB have increased expression of co-receptors for HIV, increased levels of proinflammatory cytokines, and down-regulated RANTES(Regulated upon Activation, Normal T-cell Expressed, and Secreted), which increase susceptibility to HIV infection [9]. TB could also be a sentinel diagnosis which prompted investigation for HIV in these patients. However, this suggestion cannot be concluded in our study, considering that we could not ascertain whether HIV could have preceded TB or could have been detected simultaneously with TB in some patients.

There were some limitations in our study. First, as KTBS includes only TB patients who were notified [35], the data could not reflect all TB patients in South Korea. In addition, although HIV/AIDS national registry includes all confirmed HIV/AIDS cases, there can be many undiagnosed HIV/AIDS cases. However, given that the survey conducted in PHC showed 0.08% of TB patients were diagnosed as comorbidity with HIV by HIV screening test, which is similar to the result of database data (0.07% among TB cases registered in KTBS had also be registered in HIV/AIDS registry), it was considered that our study sample could represent real life situation although there is no evidence that private and public units similarly follow the national recommendations. Second, although our survey supported the prevalence rate of TB/HIV cases from the population database data, it was not possible to compare cases within subgroups because the number of comorbid cases in the survey was too small: There were only six cases.

## Conclusions

The burden of HIV and TB/HIV is still low in South Korea, which is a low prevalent area with HIV/AIDS and a country with intermediate TB burden. However, the increase in the number of HIV patients could be a potential threat to TB control in South Korea. In addition, it is recommended that physicians should consider performing HIV tests among TB patients, especially in higher-risk groups, such as young males with extrapulmonary TB in South Korea.

## Competing interests

The authors declare that they have no competing interests.

## Authors' contributions

CHL and JGN designed the study and wrote the article. JYH and JWA analyzed the data on HIV/AIDS. JHK helped in the design of the study. EJO and HSD conducted the questionnaire survey. HJK analyzed the data on TB. MKK and SSK performed the testing on HIV. HHK and DKO supervised the study. All authors read and approved the final manuscript.

## Pre-publication history

The pre-publication history for this paper can be accessed here:

http://www.biomedcentral.com/1471-2334/10/66/prepub
